# Editorial: “*Agrobacterium* biology and its application to transgenic plant production”

**DOI:** 10.3389/fpls.2015.00265

**Published:** 2015-04-22

**Authors:** Hau-Hsuan Hwang, Stanton B. Gelvin, Erh-Min Lai

**Affiliations:** ^1^Department of Life Sciences, National Chung Hsing UniversityTaichung, Taiwan; ^2^Department of Biological Sciences, Purdue UniversityWest Lafayette, IN, USA; ^3^Institute of Plant and Microbial Biology, Academia SinicaTaipei, Taiwan

**Keywords:** *Agrobacterium*, plant genetic transformation, T-DNA, crown gall, membrane lipid, biofilm, quorum sensing, plant defense

The extraordinary *Agrobacterium* research story started from the search for the causative agent of crown gall disease more than 100 years ago. *Agrobacterium tumefaciens* was first isolated from grapevine galls in 1897 and later isolated from Paris daisy in 1907 (Cavara, 1897a,b; Smith and Townsend, 1907). The *Agrobacterium* infection mechanism involves processing and transfer of a specific DNA fragment (the transferred-DNA, T-DNA) from a bacterial tumor-inducing (Ti) plasmid. Transfer to the plant occurs via a type IV secretion system (T4SS), after which T-DNA is integrated into the plant host genome (Gelvin, 2010; Lacroix and Citovsky, 2013). This interkingdom DNA transfer leads to overproduction of the plant hormones auxin and cytokinin, resulting in tumors. The interkingdom DNA transfer ability of *Agrobacterium* and the possibility to replace the oncogenes in the T-DNA with genes of interest has made *Agrobacterium*-mediated transformation the most popular technique to generate transgenic plants.

This Research Topic provides a collection of reviews and original research articles on *Agrobacterium* genes involved in bacterial physiology/virulence and plant genes involved in transformation and defense against *Agrobacterium*. A review by Kado (2014) provides a historical overview of how *A. tumefaciens* was first established as the cause of crown gall disease. In this review, Kado highlights key early plant pathology and milestone molecular biology studies leading to the conclusion that the expression of oncogenes in native T-DNA is the cause of tumor growth in plants. With the solid foundation of these pioneering discoveries, *A. tumefaciens* evolved from a phytopathogen to a powerful genetic transformation tool for plant biology and biotechnology research.

The first complete genome sequence of an *Agrobacterium* species (*A. tumefaciens* C58) was completed in 2001 (Goodner et al., 2001; Wood et al., 2001). The 5.67-megabase genome of this strain carries one circular chromosome, one linear chromosome, and two megaplasmids: the Ti plasmid pTiC58 and a second plasmid, pAtC58. In the review by Platt et al. (2014), the properties, ecology, evolution, and complex interactions of these two *A. tumefaciens* megaplasmids are discussed. The costs and benefits to *A. tumefaciens* strains carrying the Ti plasmid and/or the pAtC58 plasmid are discussed and presented from an ecological and evolutionary perspective. Modeling predictions are presented for the relative cost and benefits to *A. tumefaciens* strains harboring the Ti and/or the pAtC58 plasmids determined by environmental resources. Conjugation and amplification of the Ti plasmid are regulated by the TraI/TraR quorum-sensing (QS) system and conjugal opines. Lang and Faure (2014) review current knowledge of the genetic networks and molecular basis of the *A. tumefaciens* quorum sensing system. These authors also discuss the biological and ecological impact of the QS system on Ti plasmid conjugation, copy number, and interactions between *Agrobacterium* and host plants.

During the initial interaction between *Agrobacterium* and plant cells, bacteria sense various plant-derived signals in the rhizosphere with the help of Ti plasmid-encoded virulence gene (*vir* gene) and chromosomal virulence gene (*chv* gene) products. The current knowledge of how *A. tumefaciens* senses and reacts to different plant-derived signals are summarized in the review article by Subramoni et al. (2014), which also discusses the mechanisms of how the plant hormones auxin, salicylic acid, and ethylene, affect bacterial virulence. Finally, this review discusses the complexity and intricacy of *Agrobacterium* signaling pathways and the underlying regulatory mechanisms during the initial host cell recognition to maximize subsequent successful infection. In the original research article by Lin et al. (2014), the mechanistic regulation of the membrane sensor VirA protein is further dissected. VirA histidine kinase and the cytoplasmic response regulator VirG protein together play a central role in regulating *vir* gene expression in response to phenolics. Based on a homology model of the VirA linker region, various mutant and chimeric VirA proteins were generated and examined for their ability to induce *VirB* promoter activity. The ability of VirA to sense and respond to three separate input signals, phenolics, sugars, and environmental pH, plays a significant role in securing successful infection.

*Agrobacterium* attachment to plant cells is an important early step in crown gall disease progression. Motile bacteria swim toward host cells and then physically interact with host cells to form aggregates and establish a multicellular bacterial community known as a biofilm. Various genetic and environmental factors that affect *Agrobacterium* attachment and biofilm formation are reviewed in the article by Heindl et al. (2014). The functions of different types of exopolysaccharides that constitute the biofilm and underlying mechanisms involving how the second messenger cyclic-di-GMP, the ChvG/ChvI system, phosphorus levels, and oxygen tension influence bacterial attachment and virulence are also summarized. In the review article by Matthysse (2014), early studies and current knowledge of the mechanisms of polar and lateral bacterial attachment are summarized. These two mechanisms both contribute to bacterial attachment. When the environmental calcium and phosphate levels and pH values are low, polar attachment predominates. In addition, the phospholipids (PLs), phosphatidylcholine (PC), and phosphate-free lipid ornithine lipids (OLs) contribute to *Agrobacterium* virulence. In the review by Aktas et al. (2014), the biosynthetic pathways and the physiological roles of these membrane lipids are summarized. The typical eukaryotic membrane lipid PC is not frequently found in bacteria, but it constitutes almost 22% of the *Agrobacterium* membrane lipid. Interestingly, PCs and OLs may play opposite roles in *Agrobacterium* virulence. The reduction of tumor formation in a PC-deficient *Agrobacterium* mutant may result from impaired *vir* gene expressions controlled by VirA/VirG. The absence of OLs in *A. tumefaciens* may decrease host defense responses and therefore cause earlier and larger tumor formation.

Plant cells have a variety of receptors that recognize so-called microbe- or pathogen-associated molecular patterns (MAMPs or PAMPs), and subsequently activate plant defense responses, a process known as Pattern-recognition receptor-Triggered Immunity (PTI) (Boller and Felix, 2009; Boller and He, 2009). *Agrobacteriium* may utilize effectors to hijack plant systems and evade plant defense responses. Pitzschke (2013) reviews strategies used by *Agrobacterium* to turn plant defense responses to its own advantage. Infected plant cells initiate a mitogen-activated protein kinase signaling cascade that causes VIP1 (*Agrobacterium* VirE2-interacting protein 1) phosphorylation and translocation into the plant nucleus to induce defense gene expression. On the other hand, *Agrobacterium* may hijack VIP1 to help T-DNA enter the plant nucleus. Based on the current knowledge of plant defense responses against *Agrobacterium* infection, Pitzschke (2013) discusses several biotechnological approaches to increase transformation efficiency. In another review by Gohlke and Deeken (2014), early plant responses to *Agrobacterium*, including various defense responses, hypersensitive responses, and phytohormone level alterations are discussed. The alterations in plant morphology, nutrient translocation, and metabolism caused by crown gall tumor formation are also reviewed. The authors summarize important genomic, epigenomic, transcriptomic, and metabolomic studies that reveal epigenetic changes associated with T-DNA integration and gall development. Subsequently, Hwang et al. (2015) review important pathogenic elicitors, host cell receptor molecules, and their downstream signal transduction pathways in host plants during the PAMP-triggered immune response. They highlight recent discoveries linking plant immunity to endomembrane trafficking and actin dynamic changes. Effects of both the host physiology, including hormone levels, circadian clock, developmental stages, and environmental factors, including light exposure lengths and temperature, on plant defense responses and bacterial virulence are reviewed and discussed.

In nature, evidence of ancient horizontal gene transfers (HGT) from *Agrobacterium* to plants has been observed in the genera *Nicotiana* and *Linaria*. Sequences homologous to mikimopine-type *Agrobacterium rhizogenes* pRiA4 T-DNA were first discovered in the genome of untransformed tree tobacco, *Nicotiana glauca*, and named “cellular T-DNA” (cT-DNA; White et al., 1983). Matveeva and Lutova (2014) review cT-DNA organization, distribution, expression regulation, and a possible correlation with genetic tumor formation in *Nicotiana* species. They also review recent findings of cT-DNA in the genomes of *Linaria* species and in other dicotyledonous families. The authors suggest that plants maintaining cT-DNA in their genomes may potentially benefit microorganisms in the rhizosphere by secreting opines in the root zone. They also propose that footprints of ancient pRi T-DNA insertions in the plant genome may provide selective advantage to these plants.

With this Research Topic we provide a platform for scientists to share their understanding of *Agrobacterium* biology and how *Agrobacterium* transforms plants. These contributions demonstrate how a highly active research community in plant and microbial sciences can elucidate important pathogenesis questions. Future research on *Agrobacteium* will continue to advance our understanding of plant-pathogen interactions, and provide new insights useful for plant genetic engineering.

## Conflict of interest statement

The authors declare that the research was conducted in the absence of any commercial or financial relationships that could be construed as a potential conflict of interest.
